# Technical Considerations in Primary Repair of a Congenital Prostatic Rectourethral Fistula in an Adult-Sized Patient

**DOI:** 10.1055/s-0041-1742155

**Published:** 2022-02-12

**Authors:** Timothy F. Tirrell, Farokh R. Demehri, Prathima Nandivada, Erin R. McNamara, Belinda Hsi Dickie

**Affiliations:** 1 Department of Surgery, Boston Children's Hospital, Boston, Massachusetts, United States; 2 Department of Urology, Boston Children's Hospital, Boston, Massachusetts, United States

**Keywords:** robotic surgery, anorectal malformation, rectourethral fistula, minimally invasive surgery

## Abstract

Congenital anorectal malformations are generally diagnosed and repaired as a neonate or infant, but repair is sometimes delayed. Considerations for operative repair change as the patient approaches full stature. We recently encountered a 17-year-old male with an unrepaired congenital rectourethral fistula and detail our experience with his repair.

We elected to utilize a combined abdominal and perineal approach, with robotic assistance for division of his rectourethral fistula and pullthrough anoplasty. Cystoscopy was used simultaneously to assure full dissection of the fistula and to minimize the risk of leaving a remnant of the original fistula (also known as a posterior urethral diverticulum).

The procedure was well tolerated without complications. His anoplasty was evaluated 60 days postoperatively and was well healed without stricture. At 9 months of follow-up, he has good fecal and urinary continence.

Robotic assistance in this procedure allowed minimal perineal dissection while ensuring precise rectourethral fistula dissection. The length of the intramural segment of the fistula was longer than anticipated.

Simultaneous cystoscopy, in conjunction with the integrated robotic fluorescence system, helped reduce the risk of leaving a remnant of the original fistula.

## Introduction

Congenital anorectal malformations are generally diagnosed and repaired as a neonate or infant. Diagnosis is sometimes missed in children with occult perineal or rectovestibular fistulae, delaying repair into adolescence and adulthood. 1 2 3 4 5 Once diagnosed, the malformation is often corrected with a standard posterior sagittal anorectoplasty (although laparoscopic assistance has been described, 5 especially for proximal fistulas).

We recently encountered a 17-year-old male patient with an unrepaired congenital rectourethral fistula. He had undergone diverting colostomy as a neonate but due to medical comorbidities, had previously been deemed too high an operative risk for repair of his rectourethral fistula. We elected to repair his fistula using a combined abdominal and perineal approach, with robotic assistance for abdominal and deep pelvic dissection. Although laparoscopic-assisted repair of anorectal malformations has been utilized for some time, 6 robotic technology is less well described and not universally available. Some reports of anorectal malformation repair using robotic systems exist, 7 8 9 but they are mostly case reports or case series—the use of the robotic system in repair of this pathology has not been studied in a broad or systematic fashion. We elected to utilize the robotic system as it afforded several advantages over laparoscopy. These advantages are presented here to describe a possible operative technique for unrepaired high anorectal malformations in adult patients.

## Method

This patient is a 17-year-old male who has a history of VACTERL. He was found to have an imperforate anus at birth and underwent diverting colostomy and mucus fistula creation on his first day of life. He had significant additional congenital malformations, including a left pulmonary artery sling and tracheal stenosis, with persistent and progressive respiratory symptoms despite attempted repair. He had frequent urinary tract infections; he strained to void and often leaked urine out of his mucus fistula. Because of his tracheal stenosis, the risk of endotracheal intubation and general anesthesia had been considered too high for repair of his imperforate anus.

**Fig. 1:**
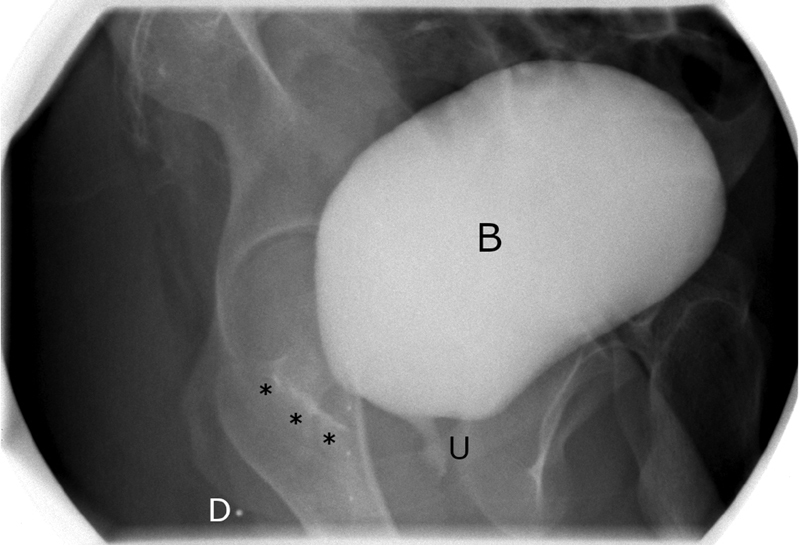
Preoperative voiding cystourethrogram. The bladder (B) is filled with contrast, which can be seen in the proximal urethra (U) and tracking posteriorly in the presumed pathway of the fistula ( *** ). Although a direct connection could not be seen on preoperative imaging, the trajectory implied a prostatic urethra origin. A radiopaque marker was placed at his external anal dimple (D) to provide an estimate of its location with respect to the fistula.

He presented to our hospital for care of his tracheal stenosis due to severe ongoing respiratory symptoms, and he ultimately underwent repeat sternotomy and a segmental tracheal resection. After recovery and stabilization of his pulmonary status, the family and patient wanted to address his rectourethral fistula due to ongoing risk of renal injury. Based on a preoperative voiding cystourethrogram, the distance between his anal dimple and the fistula was estimated to be 3 to 4 cm, and his fistula was judged to be rectoprostatic ( Fig. 1 ). Further diagnostic studies were not pursued as this information was felt to be adequate for preoperative planning and prior contrast studies performed through his mucus fistula had led to episodes of urosepsis. Because of the depth of the fistula and the extent of anticipated rectal mobilization, we elected to perform a combined abdominal and perineal approach, utilizing robotic assistance (Da Vinci Xi, Intuitive Surgical, Sunnyvale, California, United States) for takedown of his rectourethral fistula and pull-through anoplasty.

### Operative Description

**Fig. 2:**
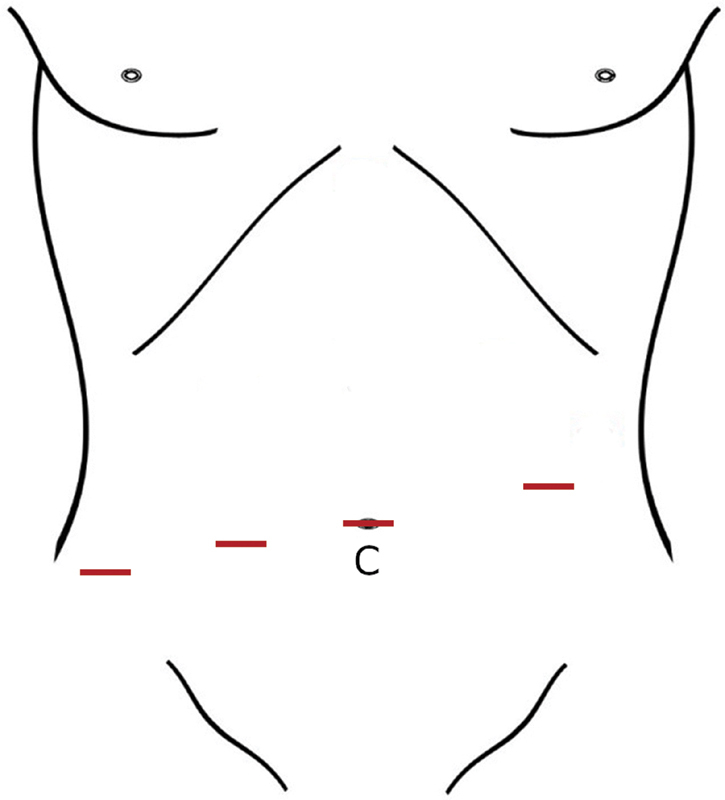
Diagram of the robotic port site placement. The camera (C) is placed at the umbilical port site and the other sites are working and assist ports. All trocars were 8 mm in diameter.

The patient was prepped and draped in low lithotomy position. The procedure started with cystoscopy that confirmed a broad-based low prostatic rectourethral fistula. A Foley catheter was placed for bladder decompression. After initially gaining umbilical access with an 8 mm trocar, three other 8 mm robotic trocars were placed in line with the umbilicus ( Fig. 2 ) oriented toward the left lower quadrant. The leftmost port was used primarily as an assistant port. Cadiere forceps were used in this port to retract the rectum out of the pelvis. The remaining two port sites generally had fenestrated bipolar forceps on the left, and a combination of curved monopolar scissors, monopolar hook cautery, and extended vessel sealer on the right. The rectosigmoid was separated from the mesorectum. Dissection was performed carefully to maintain the primary blood supply to the rectum (superior rectal artery) and to minimize injury to the rectal wall (thereby maintaining intrinsic intramural blood supply). As the dissection proceeded distally, the rectum narrowed as it approached the prostatic capsule and entered into the urethra. When approaching the fistula, dissection was performed sharply to minimize thermal spread, so we found it advantageous to place the suction irrigator on the right and the curved scissors on the left. In this orientation, the suction irrigator can be used to both clear the field of blood and bluntly retract tissue, demonstrating a plane that can be followed with sharp dissection.

Cystoscopy was repeated to confirm that we were approaching the junction with the urethra. The base of the fistula was identified cystoscopically. Using the integrated fluorescence camera, we demonstrated that there was an additional 1 to 2 cm of fistula between the base of the fistula and the extent of our intracorporeal rectal dissection. We continued the rectal dissection further and incised the common wall between the rectum and prostatic urethra. Following the fistula to its origin required opening of the prostatic capsule, and the fistula was then divided flush with the urethra under cystoscopic guidance, to avoid leaving a remnant of the original fistula (also known as a posterior urethral diverticulum) or narrowing the urethra. Visualization of the Foley catheter helped ensure that the fistula had been taken at its origin. The fistula was closed, using the robot, in two layers with running Vicryl 3–0 suture on an RB-1 needle. The urethra was filled with saline via the cystoscope and no leak was identified. The urethral catheter was replaced.

At this point, the sphincter complex was stimulated externally. A small posterior sagittal incision was made and deepened through the middle of the sphincter complex to the pelvic floor. The floor was opened and dilated to a size 24 Hegar. The rectum was then pulled into the perineal incision, which required division of some of the proximal mesenteric vessels to more easily bring it down to the perineum without tension. Visual inspection of the rectum during and after this mobilization revealed good perfusion with successful preservation of the primary blood supply. The rectum was pulled through the perineal incision; posterior rectopexy sutures to the levator muscles were placed with 3–0 Vicryl, and the anorectoplasty was completed in the usual fashion. Upon completion, the neoanus readily accommodated a 22 Hegar dilator.

## Results

**Fig. 3:**
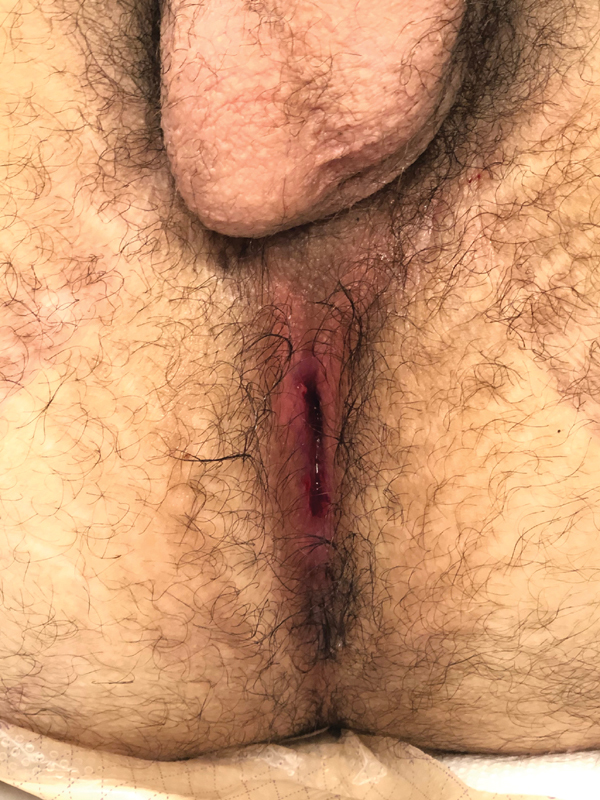
Well-healed anoplasty site, postoperative day 60.

Operative time was 387 minutes. The patient tolerated the procedure well, was extubated postoperatively, and weaned quickly to room air. He was observed overnight in the intensive care unit due to his complex airway history and transferred to the surgical floor the following day. He was discharged on postoperative day 7 (POD 7). His Foley catheter was removed on POD 14 and examination under anesthesia showed a healing anoplasty without evidence of stricture, accommodating a 16 Hegar dilator without issue. The anoplasty was again evaluated on POD 60, at the time of his colostomy closure. It was well healed without stricture and accommodated an 18 Hegar easily ( Fig. 3 ).

Due to the level of his malformation and significant amount of time he had lived without need for bowel continence, he underwent appendicostomy creation for antegrade enemas at the time of colostomy closure (approximately 2 months after the surgery described here) in anticipation of significant fecal incontinence difficulties. He utilized antegrade continence enemas for several months but at 9 months of follow-up, has achieved bowel continence and no longer needs enemas. He also has good urinary function, with some straining but good continence without leaking or accidents. He has had no further episodes of urosepsis since his surgery.

## Discussion

Repair of a congenital prostatic rectourethral fistula in an infant or neonate sometimes requires dissection of abdominopelvic structures, accomplished through laparotomy or laparoscopy. We describe the use of the robotic system and cystoscopy to repair a congenital rectourethral fistula in a full-grown patient. Utilization of the robotic system affords excellent three-dimensional visualization and the articulating instrument wrists facilitate precise instrument placement in the confined space of the deep pelvis. This simplifies both the dissection of the rectourethral common wall and the intracorporeal suturing for urethral repair. For those with access to a robotic surgery system, we advocate for its use in situations such as this.

One important recommendation in repair of a rectourethral fistula is to ligate the fistula as close to the urethra as possible, to minimize the likelihood of leaving a remnant of the original fistula (also known as a posterior urethral diverticulum) and placing the patient at risk of ongoing urinary tract infections, urinary dribbling, 10 and the potential of developing an adenocarcinoma 11 in the residual rectal tissue. In the adult-sized patient, the tissue thickness can obscure the true origin of the fistula, which we experienced here. After initial dissection, cystoscopy demonstrated that the origin of the fistula was more distal along the rectum than originally perceived based on intraabdominal evaluation. Simultaneously viewing the fistula from multiple perspectives helps ensure adequate dissection.

**Fig. 4:**
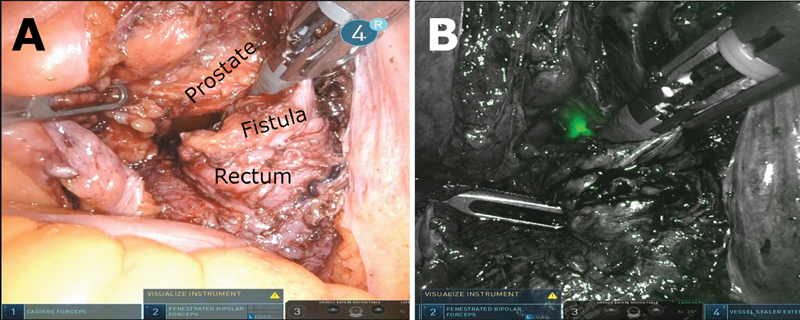
Side-by-side comparison of ( **A** ) Visual spectrum and ( **B** ) fluorescence system images. Green–red light emitted from the cystoscope is false-colored as green and easily identified.

Localizing the end of the cystoscope was facilitated by using the fluorescence system integrated into the robot. Intended to be used with a fluorophore such as indocyanine green (ICG), this system generates a composite image formed by input from three color channels generated by the image sensor. A Bayer filter is overlaid on the image sensor, dividing its sensing areas into pixels that detect red, green, and blue light. When the fluorescence system is in use, blue light is emitted from the camera and light collected by the blue pixels is used to generate the background image, which is shown in grayscale ( Fig. 4B ). Light that falls outside the spectrum of the blue pixels is displayed as green on the operator's screen. In using the system without ICG, we were able to visualize light from the cystoscope that penetrated the tissue from the urethra into the peritoneum, helping confirm the specific location of the fistula ( Fig. 4B ). Although a similar effect could be accomplished by decreasing the ambient light projected intracorporeally, the fluorescence system narrows the spectrum of the intracorporeal light, resulting in an enhanced signal to noise ratio in the green-red area of the spectrum.

In conclusion, although basic principles are unchanged, primary repair of rectourethral fistula is clearly different in an adult-sized patient than it is in a child. We find that the robotic system was very helpful for precise separation of apposed structures and for intracorporeal suturing within the constricted deep pelvic space. The intramural fistula was longer than anticipated, highlighting the need for simultaneous visual assessment of the urinary tract. To that end, the fluorescence system can be helpful in visualizing the location of the cystoscope from the abdominal viewpoint. We recognize that robotic assistance is not universally available to all practitioners but recommend its use for those who have it at their disposal.

## Notes

New Insights and the Importance for the Pediatric Surgeon Congenital anorectal malformations encountered in adults are uncommon and considerations for operative repair change as the patient approaches full stature. Robotic surgery systems can provide significant assistance in deep pelvic dissection and exact delineation of fistula anatomy.

## References

[1] Chakravartty S Maity K Ghosh D Choudhury C R Das S Successful management in neglected cases of adult anorectal malformation Singapore Med J 2009 50 08 16 18 19710959

[2] Thambidorai C R Qureshi M A Shukri J Zulfiqar A Anorectal anomalies in adult females corrected by posterior sagittal anorectoplasty Med J Malaysia 2005 60 02 226 228 16114166

[3] Tavusbay C Genç H Karaca I Atahan K Haciyanli M Türk E Successful management without protective colostomy in an adult patient with anorectal malformation Turkish J Surg. 2017 33 03 205 208 10.5152/turkjsurg.2017.3193 PMC5602314 28944335

[4] Chavan R N Chikkala B Das C Biswas S Sarkar D K Pandey S K Anorectal malformation: paediatric problem presenting in adult Case Rep Surg 2015 2015 1 4 10.1155/2015/625474 PMC4619915 26539301

[5] Miglani R K Murthy D Bhat R S Ashok K KV Anorectal anomalies in adults-laparoscopic management and review of literature Indian J Surg 2012 74 04 301 304 23904718 10.1007/s12262-011-0394-3 PMC3444603

[6] Georgeson K E Inge T H Albanese C T Laparoscopically assisted anorectal pull-through for high imperforate anus–a new technique J Pediatr Surg 2000 35 06 927 930, discussion 930–931 Available from http://www.ncbi.nlm.nih.gov/pubmed/10873037 [Internet] 10873037 10.1053/jpsu.2000.6925

[7] Albassam A Gado A Mallick M S Alnaami M Al-Shenawy W Robotic-assisted anorectal pull-through for anorectal malformations J Pediatr Surg 2011 46 09 1794 1797[Internet] 21929992 10.1016/j.jpedsurg.2011.04.019

[8] Ruiz M R Kalfa N Allal H Advantages of robot-assisted surgery in anorectal malformations: report of a case J Minim Access Surg 2016 12 02 176 178 27073314 10.4103/0972-9941.169988 PMC4810955

[9] Tseng S I Huang C W Huang T Y Robotic-assisted transanal repair of rectourethral fistula Endoscopy 2019 51 05 E96 E97 30754051 10.1055/a-0826-4220

[10] Alam S Lawal T A Peña A Sheldon C Levitt M A Acquired posterior urethral diverticulum following surgery for anorectal malformations J Pediatr Surg 2011 46 06 1231 1235[Internet] 21683228 10.1016/j.jpedsurg.2011.03.061

[11] Symons N RA Guenther T Gupta A Northover J MA Para-neorectal mucinous adenocarcinoma following childhood pull-through procedure for imperforate anus Color Dis. 2010 12 03 262 263 10.1111/j.1463-1318.2008.01762.x 19207703

